# High Prevalence of Autoimmune Diabetes and Poor Glycaemic Control among Adults in Madagascar: A Brief Report from a Humanitarian Health Campaign in Ambanja

**DOI:** 10.1155/2017/3860674

**Published:** 2017-09-06

**Authors:** Ernesto Maddaloni, Giovanlorenzo Pastore, Marco Giuseppe Del Buono, Aldostefano Porcari, Mario Fittipaldi, Francesco Garilli, Claudio Tiberti, Silvia Angeletti, Paolo Pozzilli, Giovanni Mottini, Nicola Napoli

**Affiliations:** Department of Medicine, Unit of Endocrinology, University Campus Bio-Medico, Rome, Italy

## Abstract

Madagascar is a geographically isolated country considered a biodiversity hotspot with unique genomics. Both the low-income and the geographical isolation represent risk factors for the development of diabetes. During a humanitarian health campaign conducted in Ambanja, a rural city in the northern part of Madagascar, we identified 42 adult subjects with diabetes and compared their features to 24 randomly enrolled healthy controls. 42.9% (*n* = 18) of diabetic subjects showed HbA1c values ≥ 9.0%. Unexpectedly, waist circumference and BMI were similar in people with diabetes and controls. Different from the healthy controls, diabetic subjects showed a low prevalence of obesity (5.7% versus 30%, *p* = 0.02). Accordingly, we found a high prevalence of autoimmune diabetes as 12% of people with diabetes showed positivity for the autoantibody against glutamic acid decarboxylase. Diabetic subjects with positive autoantibody had higher HbA1c values (11.3 ± 4.1% versus 8.3 ± 2.6%, *p* = 0.03) compared to diabetic subjects with negative autoantibody. In conclusion, here we describe the presence of diabetes and its features in a rural area of Northern Madagascar, documenting poor glycaemic control and a high prevalence of autoimmune diabetes. These data highlight that the diabetes epidemic involves every corner of the world possibly with different patterns and features.

## 1. Introduction

The global prevalence of type 2 diabetes is continuously increasing, and evidences show that it is no more solely a disease of affluence. Indeed, there is a call to address diabetes in the world's poorest people, where it can develop in atypical forms [1]. Moreover, the International Diabetes Federation (IDF) estimates that 80% of people with diabetes live in low- and middle-income countries. Nevertheless, in these countries, diabetes has been neglected for years and in most cases it is still undiagnosed. Data published in the 7th edition of the IDF Atlas show that 66.7% of people with diabetes living in Africa are undiagnosed [2] and thus untreated, raising concerns about the impact of diabetes and diabetes-related complications on the morbidity and mortality of African people. In this regard, we have previously shown a rising prevalence of cardiovascular complications of diabetes in rural areas of sub-Saharan Africa, highlighting the problem of cardiometabolic diseases also in the African continent [3]. Madagascar is a geographically isolated country off the southern coast of Africa, mostly medically underserved, and little is known about diabetes and its complications in this country. Because of its geographical isolation, Madagascar is considered a biodiversity hotspot with unique genomics. Both the low-income and the geographical isolation represent risk factors for the development of diabetes. In this study, we aimed to describe clinical and pathological features of diabetes mellitus in Malagasy people of Ambanja, a rural city in the northern part of Madagascar.

## 2. Methods

A clinical health campaign was conducted at the “St. Damien” Hospital in Ambanja in October 2013. 650 voluntary outpatients were tested for blood glucose to identify subjects with unknown diabetes mellitus. Diabetes mellitus was diagnosed according to ADA diagnostic criteria (at least two fasting glycaemia > 126 mg/dl or one random glycaemia > 200 mg/dl with symptoms of diabetes) [4]; subjects with fasting blood glucose between 110 mg/dl and 126 mg/dl underwent an Oral Glucose Tolerance Test (OGTT), and diagnosis of diabetes mellitus was made if blood glucose 120 minutes after the ingestion of 75 grams of glucose was >200 mg/dl.

All subjects found to be affected by diabetes were enrolled. Moreover, nondiabetic subjects were recruited at the rate of 1 control each 2 diabetic subjects (1 : 2) as controls. An additional 15% of control subjects were recruited to increase the sample size of the study. A blood sample was drawn from all enrolled subjects in order to measure HbA1c (HPLC liquid chromatography). Autoantibodies against glutamic acid decarboxylase (GADA) were measured by a radioimmunoprecipitation assay [5] utilizing a human recombinant full-length GAD65 cDNA provided by Dr Å. Lernmark (Lund University, Malmö, Sweden). The GAD cDNA was transcribed *in vitro* and translated in the presence of [35S]-methionine (Perkin-Elmer Italia, Monza, Italy) using the Sp6 TNT-coupled rabbit reticulocyte system (Promega Italia, Milan, Italy). A GADA index of 0.019 was used as a limit of positivity of the assay. It was calculated according to the 99th percentile of 200 healthy controls (GADA index = sample cpm − negative standard control cpm/positive standard control cpm − negative standard control cpm). In Diabetes Autoantibody Standardization Program (DASP IDS/CDC), the GADA assay obtained 97% specificity and 88% sensitivity. GADA were measured only in samples from subjects with diabetes. C-peptide was measured in sera from subjects with diabetes by sandwich chemiluminescent immunoassay (LIAISON, DiaSorin, Saluggia, VC, Italy).

A complete physical examination was performed: weight was recorded to the nearest 0.1 kg; height was measured with a stadiometer and recorded to the nearest 0.5 cm; body mass index (BMI) as the ratio between weight in kg and the squared height in meters; waist circumference was measured using a metric nonstretching measuring tape, midway between the inferior margin of the lowest rib and the iliac crest in the horizontal plane at the end of normal expiration and recorded to the nearest 1.0 cm; blood pressure (systolic and diastolic) was measured by trained doctors while patients were sitting.

Values are expressed as mean ± standard deviation (SD) for continuous variables and as proportions for categorical variables (%). The Shapiro-Wilk normality test was used to assess the normality of continuous variable distributions (variables with the Shapiro-Wilk statistic < 0.9, *p* values < 0.05 were considered nonnormally distributed). Comparisons between groups were done using Student's *t*-test, Kruskal-Wallis, chi-square, or Fisher's exact test depending on distribution. A two-tailed *p* value < 0.05 was considered statistically significant. All statistical analyses were performed using SPSS 21.0 for Windows.

The study was performed in accordance with the Declaration of Helsinki, and the protocol was approved by the ethic committee of the University Campus Bio-Medico of Rome.

## 3. Results

**Figure 1:**
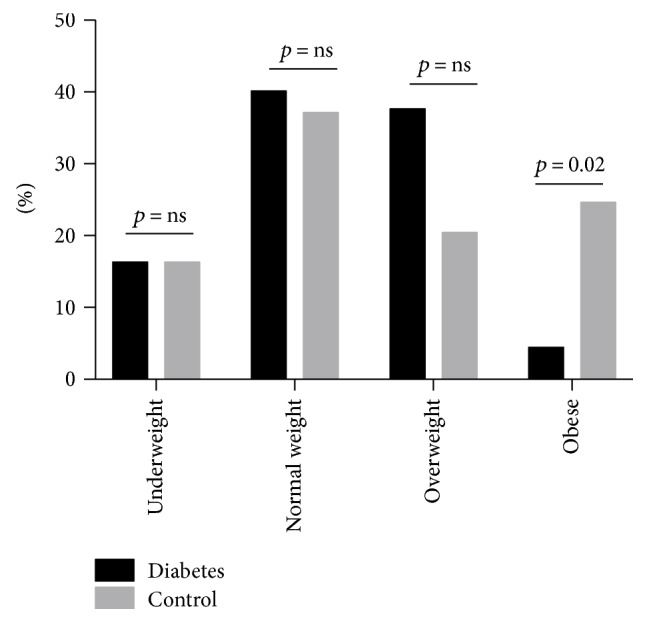
Distribution of BMI categories among diabetes and control subjects. The prevalence of obesity was significantly higher in controls compared to subjects with diabetes.

Forty-two subjects were affected by diabetes and showed higher fasting glycaemia and HbA1c than controls (*n* = 24); no significant differences were found between diabetics and controls in terms of alcohol consumption, smoking status, and family history of diabetes or obesity. Surprisingly, no significant differences were found in body mass index (BMI), waist circumference, and blood pressure between the two groups (Table 1). The distribution of BMI categories (underweight, normal weight, overweight, and obese) showed an unusual pattern, with more underweight-normal weight subjects than overweight-obese subjects (68.6% versus 51.4%). Moreover, the proportion of obese subjects among healthy individuals was significantly higher than that observed among diabetic subjects (25.0% versus 4.8%, *p* = 0.02) (Figure 1).

42.9% (*n* = 18) of subjects with diabetes showed HbA1c values ≥ 9.0%, while just 31.0% (*n* = 13) showed an adequate metabolic control, with a HbA1c value < 7.0%. Mean C-peptide levels were 2.1 ± 1.9 ng/ml. C-peptide levels showed a positive association with BMI (*r* = 0.41, *p* < 0.01).

A high prevalence of GADA positivity was found (12%) among people affected by diabetes. GADA-positive subjects had significantly higher HbA1c values (11.3% ± 4.1% versus 8.3% ± 2.6%, *p* = 0.01). Accordingly, GADA-positive subjects showed a trend towards higher levels of fasting glycaemia (298.0 ± 99.2 versus 208.8 ± 117.6, *p* = 0.06) than GADA-negative subjects (Table 2).

## 4. Discussion

In this report from a humanitarian health campaign we documented the presence of diabetes and its features in a rural area of the northern Madagascar, filling a hole in our knowledge of diabetes in southern Africa. IDF recently stated the number of data sources examining diabetes in adults in the Africa region is still very low, but no data were reported specifically for Madagascar. Our data show a poor level of metabolic control and widespread ignorance of the disease, and confirm that diabetes is a health emergency which involves also the underserved countries which are rapidly experiencing an uncontrolled westernization of diet and habits. This could have a tremendous impact in geographical regions with different and unknown genetic backgrounds, such as African countries in general and Madagascar in particular. Different populations could present different types of diabetes, with varying severity of insulin deficiency and/or resistance, and this has implications for clinical features, management and outcomes [6]. Indeed, Malagasy people living in the neighbourhood of Ambanja show different features from classical type 2 diabetes (T2D), as for waist circumference and BMI that are comparable to the healthy controls. Since we have confirmed also in this population that higher BMI is associated to higher levels of serum C-peptide, this suggests that diabetic people from Ambanja may have lower levels of insulin resistance. Moreover, more diabetics were underweight or of normal weight than overweight/obese, confirming previous observations in other low-income populations [6]. The clinical features we found could be in part be explained by our findings of a high prevalence of GADA positivity, 12% compared to those described in other countries [7–9]. Indeed, GADA-positive adults with diabetes have been shown to have lower BMI than GADA-negative subjects in previous reports [7, 9, 10]. Accordingly, we found a trend towards lower BMI in GADA-positive subjects, even though the low power of the analysis due to the low sample size is probably affecting the statistical significance. Other factors such as malnutrition and genetic background may also have an important role in this population in determining the clinical features of diabetes, and further studies in this regard are needed. Diabetes mellitus is a heterogeneous syndrome where genetic predisposition plays an important role in both T2D and autoimmune. In particular, prevalence of autoimmune diabetes appears to be very high in geographically isolated countries like Sardinia [11, 12]. Madagascar is a low-income island country in the Indian Ocean, off the southern coast of Africa. Malagasy is a unique population with different genetic background and peculiar food habits for an African population. While the poverty of urban areas increases the risk of type 2 diabetes, geographical isolation may predispose to an increased risk of autoimmune diabetes, in part explaining the high prevalence of adult autoimmune diabetes we found. As already shown in other ethnic groups [9, 10], Malagasy adults with autoimmune diabetes showed higher fasting glycaemia and HbA1c values than Ab-negative subjects. The poorer metabolic control of subjects affected by autoimmune diabetes, together with the high prevalence we described, is an alarming finding of this study, calling for urgent actions to address diabetes in this region.

We acknowledge this study has several limitations. As first, we are reporting results from a small sample size, but the study is still of value because of the geographical area where they were collected. Indeed, to the best of our knowledge, no diabetes registries were available for this area to describe diabetes in a larger cohort. Thus, we had to rely only on data collected during our humanitarian mission from people identified as being affected by diabetes. As well, methods used to screen subjects and the recruitment strategy may have partially led to a population bias. Therefore, we are not reporting data about diabetes prevalence overall and we aimed to characterize only the clinical and the pathophysiologic features of diabetes in this region. Moreover, we did not collect data about education and occupation of the enrolled subjects, which would have helped in the description of diabetes in this area. Finally, we did not investigate the presence of monogenic forms of diabetes. Because of the above-discussed geographically isolation, monogenic diabetes could also be prevalent in this area and could theoretically account for the unusual pattern of BMI distribution we found.

Nevertheless, in this study, we describe for the first time the presence and features of autoimmune diabetes of the adults in Madagascar and highlight the burden of diabetes in a corner of the world far away from the lights of medical research and healthcare systems.

## Figures and Tables

**Table 1:** Population features.

	Diabetes (*n* = 42)	Nondiabetes (*n* = 24)	*p*
Age, years (mean ± SD)	57.4 ± 11.8	62.4 ± 13.9	0.13
Sex			0.14
M, *n* (%)	20 (47.6)	7 (29.2)	
F, *n* (%)	22 (52.4)	17 (70.8)	
BMI, kg/m^2^ (mean ± SD)	23.4 ± 4.6	25.1 ± 5.3	0.16
Waist circumference, cm (mean ± SD)	83.4 ± 14.9	84.4 ± 12.0	0.79
Blood pressure			
Systolic, mmHg (mean ± SD)	144.5 ± 31.0	142.7 ± 27.0	0.81
Diastolic, mmHg (mean ± SD)	90.6 ± 14.8	89.5 ± 14.7	0.79
Smoke, *n* (%)	5 (11.9)	2 (8.3)	0.50
Alcohol, *n* (%)	6 (14.3)	3 (12.5)	0.58
Family history of DM, *n* (%)	14 (33.3)	5 (21.7)	0.25
Family history of obesity, *n* (%)	16 (38.1)	9 (37.5)	0.59
Fasting glycaemia, mg/dl (mean ± SD)	219.4 ± 118.2	104.2 ± 19.7	<0.01
HbA1c, % (mean ± SD)	8.6 ± 2.9	5.8 ± 0.7	<0.01

**Table 2:** Population features by GADA positivity.

	GAD+ (*n* = 5)	GAD− (*n* = 37)	*p*
Age, years (mean ± SD)	56.0 ± 8.5	57.6 ± 12.3	0.78
Sex			0.55
M, *n* (%)	2 (40.0)	18 (42.9)	
F, *n* (%)	3 (60.0)	19 (45.2)	
BMI, kg/m^2^ (mean ± SD)	20.1 ± 1.8	23.8 ± 4.6	0.09
Waist circumference, cm (mean ± SD)	73.2 ± 15.5	84.8 ± 14.4	0.10
Blood pressure			
Systolic, mmHg (mean ± SD)	119.2 ± 19.8	148.1 ± 30.8	0.05
Diastolic, mmHg (mean ± SD)	79.4 ± 11.0	91.4 ± 15.1	0.37
Smoke, *n* (%)	0 (0)	5 (11.9)	0.55
Alcohol, *n* (%)	2 (40)	4 (9.5)	0.14
Family history of DM, *n* (%)	2 (40)	12 (28.6)	0.55
Family history of obesity, *n* (%)	2 (40)	14 (33.3)	0.64
Fasting glycaemia, mg/dl (mean ± SD)	298.0 ± 99.2	208.8 ± 117.6	0.06
HbA1c, % (mean ± SD)	11.3 ± 4.1	8.3 ± 2.6	0.03
C-peptide, ng/ml (mean ± SD)	1.5 ± 1.1	2.2 ± 2.0	0.91
